# Genomic insights into the coupling of a *Chlorella*-like microeukaryote and sulfur bacteria in the chemocline of permanently stratified Lake Cadagno

**DOI:** 10.1038/s41396-023-01396-y

**Published:** 2023-04-08

**Authors:** Jaspreet S. Saini, Mosè Manni, Christel Hassler, Rachel N. Cable, Melissa B. Duhaime, Evgeny M. Zdobnov

**Affiliations:** 1 Department F.-A Forel for Environmental and Aquatic Sciences, Earth and Environmental Sciences, University of Geneva, Geneva, Switzerland; 2 Department of Genetic Medicine and Development, University of Geneva, Geneva, Switzerland; 3 Swiss Institute of Bioinformatics, Lausanne, Switzerland; 4 Institute of Earth Sciences, University of Lausanne, Lausanne, Switzerland; 5 Department of Ecology and Evolutionary Biology, University of Michigan, Ann Arbor, MI USA; 6 Present Address: Laboratory for Environmental Biotechnology, Ecole Polytechnique Fédérale de Lausanne, Lausanne, Switzerland

**Keywords:** Microbial ecology, Metagenomics

## Abstract

Meromictic Lake Cadagno is a permanently stratified system with a persistent microbial bloom within the oxic-anoxic boundary called the chemocline. The association between oxygenic and anoxygenic photosynthesis within the chemocline has been known for at least two decades. Although anoxygenic purple and green sulfur bacteria have been well studied, reports on oxygenic phytoplankton have remained sparse since their discovery in the 1920s. Nearly a century later, this study presents the first near-complete genome of a photosynthetic microbial eukaryote from the chemocline of Lake Cadagno, provisionally named *Chlorella*-like MAG. The 18.9 Mbp nuclear genome displays a high GC content (71.5%), and the phylogenetic placement suggests that it is a novel species of the genus *Chlorella* of Chlorophytes. Functional annotation of the *Chlorella*-like metagenome-assembled genome predicted 10,732 protein-coding genes, with an approximate 0.6% proportion potentially involved in carbon, sulfur, and nitrogen (C, N, and S) metabolism. In addition to C4 photosynthesis, this study detected genes for heat shock proteins (HSPs) in the *Chlorella*-like algae, consistent with the other *Chlorella* species. Altogether, the genomic insights in this study suggest the cooperation of photosynthetic algae with phototrophic sulfur bacteria via C, N, and S metabolism, which may aid their collective persistence in the Lake Cadagno chemocline. Furthermore, this work additionally presents the chloroplast genome of *Cryptomonas*-like species, which was likely to be presumed as cyanobacteria in previous studies because of the presence of phycobilisomes.

## Introduction

Meromictic Lake Cadagno is situated at an altitude of 1921 m within the Swiss Alps, with permanent stratification into three zones: mixolimnion (upper oxic), monimolimnion (lower anoxic), and chemocline (oxic-anoxic boundary) [1–6]. The chemocline harbors a persistent microbial bloom that coincides with decreased oxygen and light concentrations but increased ammonium, iron, and sulfide concentrations [7–13]. Within these physicochemical changes of the chemocline, Chl *a* and turbidity peaks have been used as a proxies for oxygenic phytoplankton (Chl a) and anoxygenic photo- and chemotrophic sulfur bacteria (turbidity) for at least two decades [14–16]. Oxygenic photosynthesis specifically facilitates dark aerobic sulfide oxidation [17] by *Chromatium okenii*, a purple sulfur bacterium, which is also known to contribute most to the biomass of Lake Cadagno chemocline [1, 18]. Oxygenic photosynthesis also fuels in situ oxygen for methane oxidation [19] and may create microaerophilic conditions for iron oxidation [20]. Since in situ oxygen production is proposed to be essential for methane oxidation, iron oxidation, and dark aerobic sulfide oxidation in the chemocline, identifying oxygen-producing phototrophs, including photosynthetic algae and diatoms, remains limited to microscopy [17, 19].

Phycobilin-containing cells and phycocyanin signals are often used as proxies for cyanobacteria in Lake Cadagno (June and October 2013 [21], August [12] and September 2017 [22], and August 2019 [15]) and were hypothesized to create microoxic conditions by facilitating in situ oxygen production [12, 22]. However, information on cyanobacterial species contributing to phycobilin and phycocyanin signals was lacking. While these studies used flow cytometry to identify cyanobacteria, a parallel 16S amplicon gene phylogeny from samples collected in August 2017 revealed that cyanobacteria were rare and that the chloroplasts of Chlorophyta (green algae) and Ochrophyta species (diatoms) were abundant in the chemocline [16]. These contrasting findings on cyanobacteria and chloroplasts warrant the genomic characterization of the microbial communities present in the chemocline of Lake Cadagno, as both can perform oxygenic photosynthesis.

The photosynthetic pigments (chlorophyll *a* and phycocyanin) of oxygenic phototrophs or phytoplankton peak at the beginning of the chemocline [7, 14, 15], where particulate sulfur, hydrogen sulfide, particulate organic nitrogen, and ammonium concentrations also start to rise [16]. Sulfide is toxic to most eukaryotes, but algae may acquire sulfur from sulfate [23], and some microalgae, such as *Chlorella sorokiniana*, are also capable of reducing sulfide to sulfate [24]. In addition to sulfur, some reports have highlighted that microbial eukaryotes [25] and cyanobacteria [26] can also metabolize nitrogen. However, their prospective roles in sulfur and nitrogen cycling in Lake Cadagno remain unknown.

The prokaryotic population of Lake Cadagno has been rigorously studied, and the genomes of anoxygenic purple sulfur bacteria (for example, *C. okenii* and *Thiodictyon syntrophicum*) that modulate sulfur and nitrogen metabolism have been uncovered [11, 27]. No microbial eukaryotic genome has been described for the Lake Cadagno water column since its initial observation in the 1920s [28]. This study presents the first near-complete genome of the most abundant photosynthetic microbial eukaryote at the beginning of the Lake Cadagno chemocline, focusing on its potential for carbon, sulfur, and nitrogen (C, N, and S) metabolism.

## Results and discussion

Phytoplankton, and phototrophic sulfur bacteria peak in the oxic-anoxic boundary called the chemocline of Lake Cadagno, as indicated by photosynthetic pigments (chlorophyll a and phycocyanin) and turbidity (13–15.5 m, Fig. S1) [16]. These microbial peaks distinguish upper-oxic mixolimnion from lower anoxic-monimolimnion, and shotgun DNA sequencing in this study investigated these communities following prior hypotheses of in situ oxygen production by photosynthetic algae [17, 19], and cyanobacteria [22].

### Protist community composition and reconstruction of algae and diatoms genomes

**Fig. 1:**
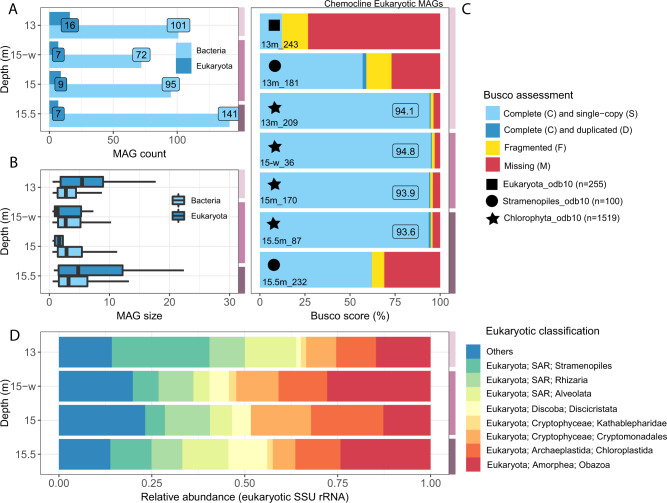
Overview of four metagenomes collected between 13–15.5 m depth of Lake Cadagno’s chemocline, where 15-w represents a whole water sample (without 55 µm mesh). **A** Bacterial and eukaryotic MAGs obtained through competitive binning via CONCOCT. **B** Size of MAGs classified by CAT/BAT taxonomy (hits >0.5 bit-scores). **C** Quality assessment of MAGs using BUSCO (only eukaryotic MAGs with >10% completeness are showed). **D** Eukaryotic community classification using 18S rRNA gene sequences extracted from the libraries with Phyloflash. The light-lilac, lilac, and dark-lilac color columns on the right y-axis of the plots indicate the mixolimnion-chemocline transition, chemocline, and lower chemocline, respectively.

From the four chemocline samples, millions of raw reads were quality checked, normalized (29,747,969–80,546,876; Table S1), and assembled into contigs using SPAdes. The total size of assembled contigs ranged between 360–890 Mbp with N50 values between 3,496–8,393 bp and a total number of contigs between 87,929–232,398 (Fig. S2A–D). At 15 m depth, where phytoplankton (Chl *a*, phycocyanin) were close to the maximum, 7,659 contigs (L50) contributed to 50% of the whole metagenome assembly with a minimum length of 8,393 bp (N50), indicating the contribution of longer contigs (Fig. S2C; 15-w). From each depth of the chemocline, hundreds of Metagenome-Assembled Genomes (MAGs) were obtained based on the coverage and sequence composition using CONCOCT (Fig. 1A) [29]. Most of the resulting MAGs belonged to bacterial lineages, with only a few microbial eukaryotic genomes (4.7 to 13.6% of total MAGs; Fig. 1A, B). This low occurrence of eukaryotic MAGs may be due to their low abundance and may also reflect the challenges associated with eukaryotic genome binning owing to their larger genome size than most prokaryotes observed in the Lake Cadagno chemocline (Fig. 1B, C) [30]. MAGs of phytoplankton, including cyanobacteria and photosynthetic microbial eukaryotes, have been further scrutinized for their involvement in in situ oxygen production via oxygenic photosynthesis [17, 19, 22]. Typical cyanobacteria-specific phycocyanin and phycobilin signals have been reported (years 2013 [21], 2017 [12, 22], and 2019 [15]) for the chemocline of Lake Cadagno. In contrast, this study recovered only one putative cyanobacteria-like MAG with 11.4% completion, based on marker genes detected by BUSCO (Benchmarking Universal Single-Copy Orthologs) (13m_Bin_1, Table S2), which may be due to their previously observed low abundance in the chemocline [16].

By analyzing metagenomics reads for 18S rRNA genes, this study observed a mixed population of microbial eukaryotes (Fig. 1D). The relative abundance of eukaryotic community composition in the chemocline was as follows: Alveolata (4.3–13.8%), Cryptomonadales (6.0–16.2%), Obozoa (12.7–27.8%), Rhizaria (8.3–12.1 %), Chloroplastida (10.7–19.5%) and Stramenopiles (5.3–26.2%). Previously, the relative abundance (%) based on 18 S rRNA gene sequences indicated the dominance (>70%) of Cryptophyta with approximately 5–10% of Perkinsozoa (Alveolata) and Stramenopiles [6]. Dinoflagellates (Alveolata), cryptophytes (Cryptomonadales), chlorophytes (Chloroplastida), and diatoms (Stramenopiles) contain chlorophyll a (Chl *a*) [31], which may contribute to oxygenic photosynthesis in the chemocline. However, genus or species-level investigations are required to know the identity of microorganisms, and eukaryotic MAGs provide further insights.

From the overall eukaryotic populations, BUSCO in the auto-lineage mode identified eukaryotic MAGs with a wide range of gene content completeness (38–94.8%), including Chlorophyta (algae; genus *Chlorella*, up to 94.8% completeness) and Stramenopile (diatom; genus *Nitzschia*, up to 62% completeness), when using both competitive (Fig. 1C; Table S2), and non-competitive binning (Fig. S2G; Table S3). Genomes from these photosynthetic clades were expected because chloroplast amplicons of Chlorophyta and Ochrophyta (Stramenopile) were identified in a previous study [16]. Putative MAGs of other eukaryotic algae (Cryptophyceae), including the genus *Cryptomonas* and *Gullardia*, were also obtained but had low completion (<10%, Table S10). Overall, these results provide the first assembled genomes of algae and diatoms from the chemocline of Lake Cadagno, which have the potential to contribute to oxygenic photosynthesis.

### High-quality eukaryotic genome of a novel species of *Chlorella*-like microorganism

**Fig. 2:**
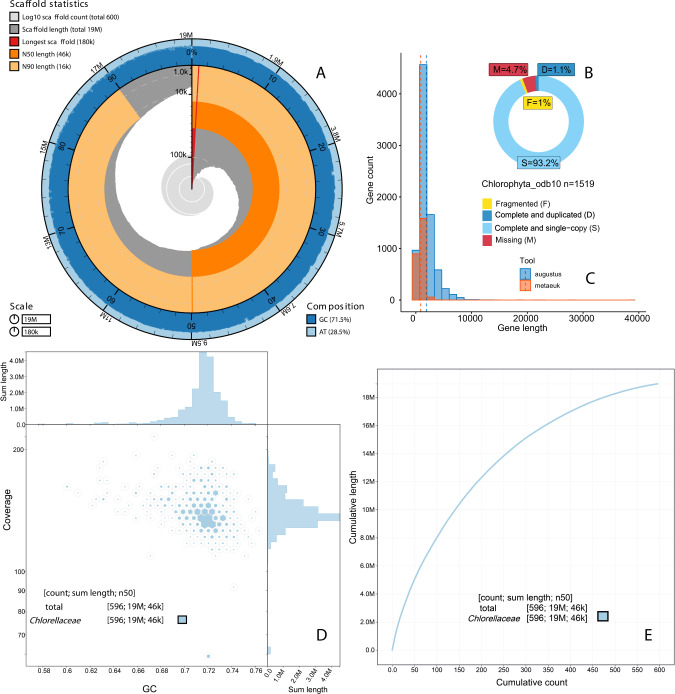
Statistics on the newly assembled Lake Cadagno MAG visualized using Blobtools and R-studio. **A** Snail plot of the 18.97 Mbp Chlorophyta genome assembly (Maximum contig length 181 Kbp, shown in red). Orange and pale-orange arcs indicate the N50 and N90 values, respectively, where the longest scaffold is displayed in red. **B** Eukaryotic genome quality assessment by BUSCO using Chlorophyta dataset (*n* = 1519). **C** The number of protein-coding genes predicted in Chlorophyta MAG by EukMetaSanity based on Augustus and MetaEuk gene predictors. **D** Hexagon-binned plot of GC content and coverage based on mapping with Chlorophyta-specific raw reads obtained from the chemocline samples (13, 15-w, 15, and 15.5 m). **E** Taxonomic classification of Lake Cadagno MAG contigs calculated using the Diamond BLASTx nr database (updated 24 July 2021).

More genomes of protists are needed to advance our understanding of their biology [32, 33]; however, obtaining well-curated microbial eukaryotic genomes from metagenomes remains challenging owing to their large genome size and complexity [30, 34]. Although there are pioneering studies in other systems [35, 36], there have been no prior reports on eukaryotic genomics from the Lake Cadagno chemocline. In this study, after co-assembling the Chlorophyta-specific reads, a representative Chlorophyta genome of 19.4 Mb (636 contigs; minimum length: 2500 bp, N50: 45 Kbp, Table S4) was obtained by re-assembling and re-binning using SPAdes and CONCOCT respectively. From this representative genome, prospective contaminant contigs (*n* = 40, Table S5) were removed by referring to GC content and coverage using the Anvi’o interface guided by CAT taxonomy. After removing the contaminants, a final 18.9 Mbp-genome with 596 contigs with a maximum contig length of 181 Kbp was obtained (N50: 46 Kbp, Table S6). The refined Chlorophyta MAG had 93.2% BUSCO completion (*n* = 1519, Chlorophyta dataset), with 10,732 protein-coding genes and an average genome coverage of 141X (Fig. 2A–C). Not only did the rebinning and refining step result in a decrease in the total number of contigs, but the contribution of long contigs also increased (total contig = 596, of which 19 > 100 Kbp, 119 > 50 Kbp, and 282 > 25 Kbp) compared to the best-quality MAG obtained during primary competitive binning (15-w_36, total contig = 1104, of which 3 > 100 Kbp, 49 > 50 Kbp, and 257 > 25 Kbp). However, the BUSCO completeness score of the refined MAG slightly decreased (by 1.6%) compared with that before refinement MAG (15-w_36, 94.8% complete; Fig. 1C).

**Fig. 3:**
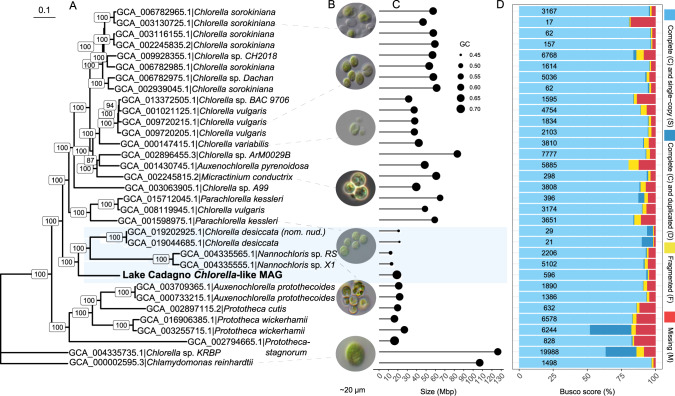
Comparative analyses of Lake Cadagno *Chlorella*-like MAG with relatives from the Chlorellaceae family (NCBI ID 35461). **A** Phylogenomic tree of *Chlorella*-like MAG with other species from the Chlorellaceae family (NCBI TaxID: 35461). The maximum likelihood phylogeny was estimated from a super-alignment (43,679 aa) of 95 single-copy orthologs, using *Chlamydomonas reinhardtii* as the outgroup species. Branch length represents the substitutions per site. Values on the nodes indicate bootstrap support. **B** Pictures of *Chlorella* species obtained from the culture collection of algae and protozoa (CCAP; https://www.ccap.ac.uk). **C** Size and GC content comparison. **D** BUSCO quality assessment using the Chlorophyta_odb10 dataset (number of markers = 1519). The values displayed on the bars represent the number of contigs in the genome assembly.

The taxonomic classification with contig annotation tool (CAT) and the diamond BLASTx results against the nr database indicated that this MAG belonged to Chlorellaceae family (Table S2, Fig. 2E). The phylogenomic analysis also placed it close to other *Chlorella* species (Fig. 3A). Hence, we provisionally refer to this newly assembled genome as *Chlorella*-like MAG. It is likely that this *Chlorella*-like species belongs to an early branching lineage of small microbial eukaryotes such as *Nannochloris* and *Chlorella desiccata*, but had a significantly higher GC content when compared to the closest relatives (Fig. 3A–C; 71.50 vs. 40–45). Microscopic images of the closest relatives (species from Chlorellaceae) indicated that the *Chlorella*-like MAG belonged to the nanophytoplankton community (2–20 µm) [37]. The *Chlorella*-like assembly exhibited high genome quality (93.2% single copy, 1.1% duplicated, 4.7% missing, and 1.0% fragmented) compared to the mean completeness of Chlorellaceae assemblies available at NCBI (87.9%, Fig. 3D).

The predicted proteome from the *Chlorella*-like MAG was mapped to the OrthoDB v10.1 [38] online database at the Trebouxiophyceae level (Chlorophyta Class) which includes 5 species: *Auxenochlorella protothecoides* (GCF_000733215.1), *C. sorokiniana* (GCA_002245835.2), *Chlorella variabilis* (GCF_000147415.1), *Coccomyxa subellipsoidea* C-169 (GCF_000258705.1), and *Helicosporidium sp*. ATCC 50920, (GCA_000690575.1). Approximately 6,000 protein-coding genes from the *Chlorella*-like MAG had orthologs in at least one of the other five species (Fig. S3). From the chemocline of the meromictic Lake Cadagno, these findings represent the first extensively curated high-quality eukaryotic genome and its predicted proteome.

### Genomes of *Chlorella*-like and *Cryptomonas*-like chloroplasts in the chemocline

**Fig. 4:**
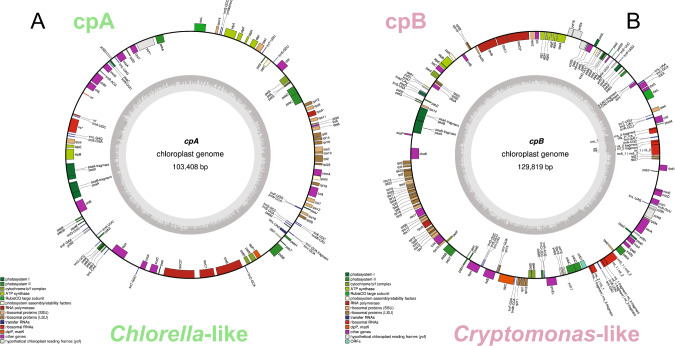
GeSeq based annotation of chloroplasts genomes detected in the chemocline of Lake Cadagno. **A** Chloroplast of *Chlorella*-like (cpA) algae with the genome size of 103 Kbp. **B** Chloroplast of *Cryptomonas*-like (cpA) algae with the genome size of 130 Kbp.

**Fig. 5:**
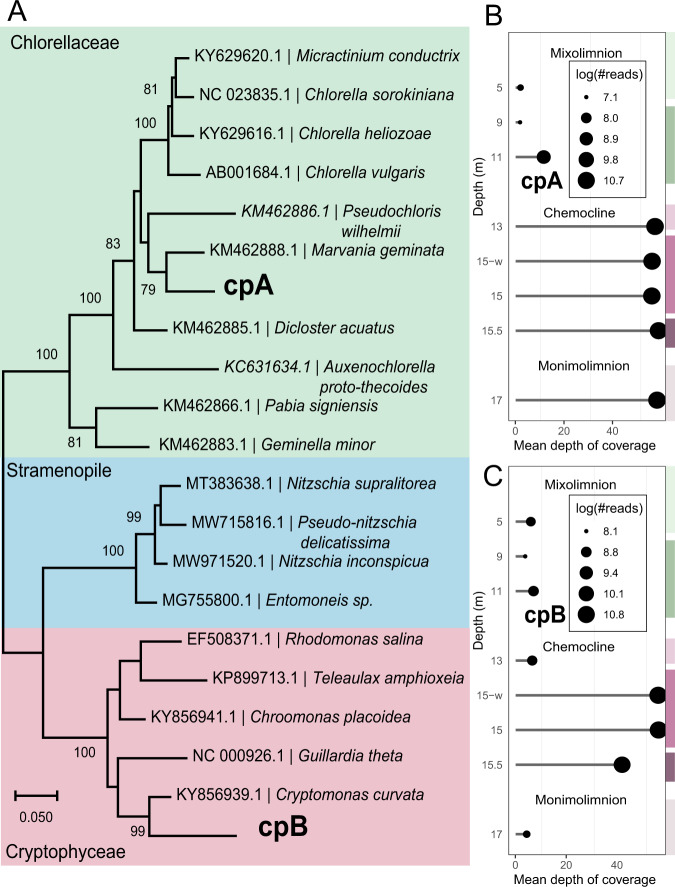
Phylogenomic representation of the chloroplast genomes of *Chlorella-like* and *Cryptomonas-like* algae assembled using NOVOPlasty. **A** Phylogenetic tree based on 18 chloroplast marker genes. Bootstrap support values are shown on nodes. **B**, **C** Chloroplast coverage and the number of reads (log) were calculated throughout the water column using Bowtie 2 and SAMtools. The light-green, dark-green, light-lilac, lilac, dark-lilac, and light-brown columns on the right y-axis indicate the zones of high-O_2_ mixolimnion, medium-O_2_ mixolimnion, mixolimnion-chemocline transition zone, chemocline, lower anoxic chemocline, and monimolimnion, respectively.

Previous studies have reported the possible presence of cyanobacteria in the chemocline of Lake Cadagno based on phycocyanin and phycobilin signals [12, 21, 22]; however, a 16S amplicon gene phylogenetic study limited to amplicon data identified chloroplasts [16]. Here, the metagenomic dataset provides evidence for Cryptophyceae (*Cryptomonas curvata* and *Guillard theta*) with 9 putative MAGs from the chemocline samples (Table S2). *Cryptomonas* are known for their phycobiliproteins, two of which (phycocyanobilin and phycoerythrobilin) are present in cyanobacteria [39]. The putative cyanobacterial-like MAG detected in Lake Cadagno was classified as *C. curvata* using the contig annotation tool (CAT) (Table S2). The phycobiliproteins are located at the thylakoid lumen of *Cryptomonas* chloroplast [40], and *Cryptomonas* have also been previously identified in Lake Cadagno [6, 14]. Thus, in addition to identifying the chloroplasts of newly identified *Chlorella*-like species, the *Cryptomonas* chloroplasts were also targeted. Using the available chloroplast genomes of Chlorellaceae (*Parachlorella kessleri*; NC_012978.1) and Cryptophyceae (*G. theta*; NC_000926.1) from NCBI, BLASTn identified two prospective contigs with a size of at least 100 Kbp, here referred to as Chloroplast A (cpA) and Chloroplast B (cpB) (Fig. 4A, B). Followed by the circularization of cpA and cpB using NOVOPlasty, phylogenetics analysis confirmed that these prospective chloroplasts belonged to the Chlorellaceae and Cryptophyceae (Fig. 5A).

**Fig. 6:**
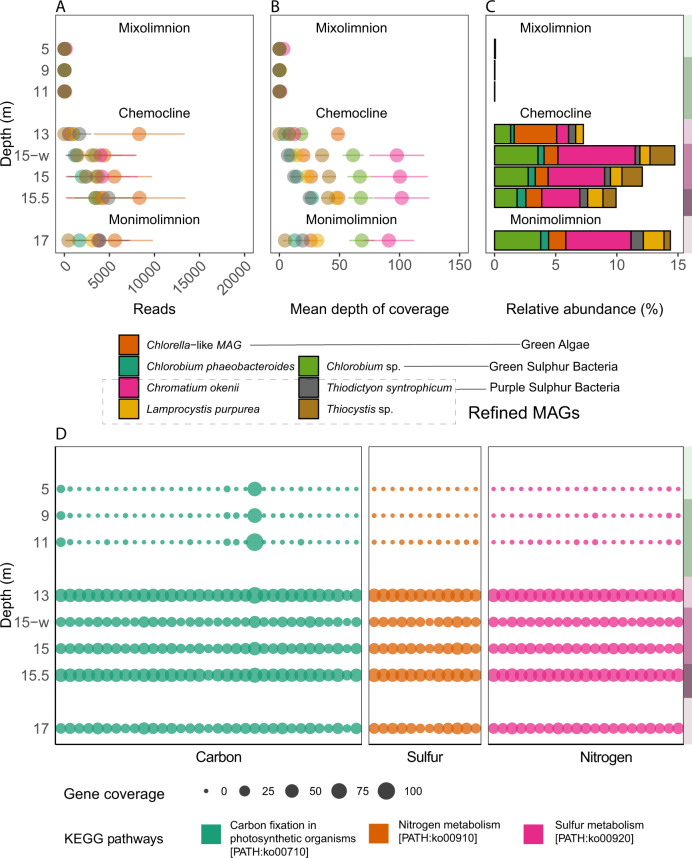
Estimation of abundance and functional annotations of Lake Cadagno *Chlorella*-like microbial eukaryotes with reference to Lake Cadagno depths. **A**–**C** Comparison of the abundance of *Chlorella*-like eukaryotic algae with purple and green sulfur bacteria based on mapping statistics: **A** number of reads mapped, **B** mean depth of coverage and **C** genome’s relative abundance (%) based on mapping using the total metagenomic reads. **D** Coverage of protein-coding genes involved in C, N, and S metabolism in the *Chlorella*-like algae. Protein-coding genes were predicted by EukMetaSanity and mapped to KEGG pathways using eggNOG. **A**–**D** The light-green, dark-green, light-lilac, lilac, dark-lilac, and light-brown columns on the right y-axis indicate the zones of high-O_2_ mixolimnion, medium-O_2_ mixolimnion, mixolimnion–chemocline transition zone, chemocline, lower anoxic chemocline, and monimolimnion, respectively.

*Chlorella*-like chloroplast (cpA) coverage and the number of reads (log) peaked in the chemocline and persisted in the monimolimnion, a pattern also matching the nuclear genome (Fig. 5B, Fig. 6A–C). Comparing the mean coverage depths of the nuclear and chloroplast genome within the chemocline (48.6 vs 55.6X at 13 m, Fig. 5B, Fig. 6B), each *Chlorella*-like cell is likely to have a single copy of the chloroplast genome and thus one chloroplast. A single chloroplast was also observed in *Chlorella protothecoides* using microscopy [41].

*Cryptomonas*-like chloroplast (cpB) coverage and read patterns coincided with phycobilisome-containing cells that peaked in the chemocline for sampling season (August 2017) [16] as this study (Fig. 5C). The genes coding for these photosynthetic reaction centers (PSI *psa* and PSII *psb*) exist in cpA and cpB chloroplasts (Fig. 4A, B). However, the phycobilisome-specific phycoerythrin protein (*cpeB*) has only been found in *Cryptomonas*-like chloroplast, and phycoerythrin has also been identified in *Cryptomonas* in Lake Cadagno [14]. In previous studies, Cyanobacteria in Lake Cadagno have been identified by targeting phycocyanins in phycobilisomes [12, 21, 22]. In contrast, this study did not recover phycocyanin genes in the *Cryptomonas*-like chloroplasts, although phycoerythrin is attached to the phycocyanins and is part of the overall phycobilisome structure [42–45]. Overall, this genomics evidence on MAGs [12, 21, 22] and chloroplast suggests that peaks of phycobilisome-containing cells by flow cytometry [12, 21, 22] and phycocyanin signals [15, 16] in chemocline may have been sourced from *Cryptomonas*-like cells.

The persistence of *Chlorella*-like algae and their chloroplasts provides new evidence of ongoing oxygenic photosynthesis in the bottom-monimolimnion. This scenario has also been proposed before for chemocline [19], where a limited amount of light may still be available for oxygenic photosynthesis. The occurrence of *Chlorella*-like eukaryotic phototrophs in the monimolimnion may also be due to sinking particles in the lower layers of the lake. However, if this scenario is true, this study would expect *Cryptomonas*-like chloroplasts to sink. Still, their read and coverage patterns decrease in monimolimnion contrary to the *Chlorella*-like algae and suggest their abundance is restricted to chemocline (Fig. 5B, C). Taken together, the phylogenetic tree, coverage, and read pattern provide evidence that both *Chlorella*-like and *Cryptomonas*-like algae may synergistically contribute to oxygenic photosynthesis in the chemocline. However, *Chlorella*-like eukaryotic algae may also persist in dark monimolimnion, where hydrogen sulfide and ammonia concentrations are at their maximum.

### Metabolic potential of *Chlorella*-like algae with the focus on C, N, and S pathways

The contributions of purple sulfur bacteria (*C. okenii*, *Lamprocystis purpurea*, *T. syntrophicum*, and *Thiocystis* sp.) and green sulfur bacteria (*Chlorobium phaeobacteroides*, *Chlorobium* sp.) for carbon, sulfur, and nitrogen metabolism in the chemocline of Lake Cadagno have been rigorously studied [1, 11, 14, 15, 17, 21, 22, 27, 46, 47]. In contrast, direct evidence of eukaryotic metabolism is yet to be established for the Lake Cadagno chemocline. New *Chlorella*-like MAG had higher read counts, coverage (48.6X), and relative abundance (3.4%) at the beginning of the chemocline than the purple sulfur bacteria (PSB) and green sulfur bacteria (GSB) (Fig. 6A–C at 13 m). PSB *C. okenii* and GSB *Chlorobium* sp. coverage (100 and 61X) and relative abundance (6.3 and 3.5%) were maximum at 15 m whole water sample from the turbidity peak (15-w, Fig. 6B, C). The increased coverage of *Chlorella*-like green algae, followed by the dominance of *Chromatium* and *Chlorobium* is expected as phytoplankton tend to stay above the phototrophic sulfur bacteria, as observed by the peak of phycocyanin, Chl *a*, and turbidity.

**Fig. 7:**
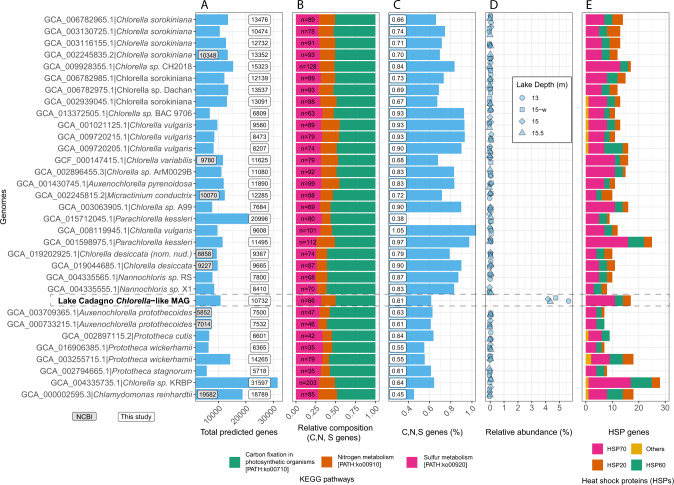
Comparison of the Lake Cadagno *Chlorella*-like MAG with other known *Chlorella* genomes. **A** the total number of protein-coding genes predicted in the present study (number of genes from the official annotation available in NCBI are shown when available); **B** Relative composition of C, N, and S genes. Values on the barplot represent the number of C, N, and S genes; **C** Percentage of C, N, and S genes among the total predicted genes; **D** Relative abundance of *Chlorella* genomes in the chemocline of Lake Cadagno. **E** The number and types of heat shock proteins identified in each genome. Only genes belonging to the Chlorophyta and Viridiplantae taxonomy were used.

Functional annotations of *Chlorella*-like MAG using OrthoLoger (Table S7) and eggNOG (Table S8) mappers revealed a repertoire of genes potentially involved in carbon [PATH:ko00710], nitrogen [PATH:ko00910], and sulfur metabolism [PATH:ko00920], with higher coverage in the chemocline and monimolimnion than in the mixolimnion (Fig. 6D; Table S9). The C, N, and S genes for *Chlorella*-like MAG constituted approximately 0.6% (*n* = 66) of the total predicted genes (*n* = 10,732) (Fig. 7A, B). The majority of C, N, and S genes belonged to carbon fixation (50%), followed by sulfur (31%) and nitrogen (18%) metabolism (Fig. 7A, B). Although this study did not detect pathways particularly enriched for *Chlorella-*like MAG, the C, N, and S metabolism, on the other hand, is consistent with the other *Chlorella* species (Fig. 7B, C). Yet, the relative abundance comparison indicates that other *Chlorella* species were not present in the chemocline (Fig. 7D). Further investigation on carbon metabolism (Figs. 6D,  7B; PATH:ko00710) identifies near-complete pathways for the C4 dicarboxylic cycle and crassulacean acid metabolism (CAM) (Fig. S4). These carbon fixation metabolisms are specialized to uptake CO_2_ in the dark and increase the CO_2_ availability for photosynthesis [48, 49]. Some genes involved in C4 photosynthesis are shown for *Chlorella variabilis*; surprisingly, they are not well described for *Chlorella* species but have been identified in other Chlorophyta genomes [50]. The functional annotations of *Chlorella* species in this study suggest genes for C4 photosynthesis are consistently present within the Chlorellaceae family (Fig. 7B; PATH:ko00710, Tables S7, S8). Under limited light in the chemocline of Lake Cadagno, such specialized carbon fixation pathways may be used for in situ oxygen production by *Chlorella*-like photosynthetic algae, which is also coupled with the dark aerobic sulfide oxidation of *C*. *okenii* [17]. The sulfur metabolic genes [PATH:00920: Table S9] in *Chlorella*-like algae may preferentially acquire sulfate [51] resulting from sulfide oxidation by *C*. *okenii* and explain its coupling with phototrophic sulfur bacteria in the chemocline (Fig. 6A–C). Genes involved in nitrogen metabolism [PATH:00910: Table S9] may be used for biomass synthesis, as it has been shown in other microalgae, including *C. sorokiniana* [52], *Chlorella vulgaris*, and *Chlamydomonas* [53–55]. Nitrate storage in microbial eukaryotes has been suggested to facilitate survival under anoxic conditions [56], supporting the potential ability of *Chlorella*-like microorganisms to persist in the anoxic zones of Lake Cadagno.

Additionally, the *Chlorella*-like microbial genome contains 17 genes belonging to heat shock protein (HSP) families consistent with other *Chlorella* species (Fig. 7E). Compared to other HSPs in the chemocline, *HSP70* has a relatively higher number of genes (Fig. 7E). The HSPs are known for participating in environmental stress response [57], including extreme temperature [58] and exposure to redox metals [57, 59], as shown for *Chlorella* [60] and other algae [57, 58, 61]. Such environmental stress might frequently occur in Lake Cadagno microbial bloom, owing to the internal oscillations of the chemocline bringing fluctuations in the temperature, light, sulfur, ammonia, and trace metal (iron and manganese) concentrations [9, 16, 62]. Thus, the abundance of heat shock proteins may aid Lake Cadagno *Chlorella* in thriving in the chemocline while maintaining cooperation with phototrophic sulfur bacteria via C, N, and S metabolism.

## Conclusion

Microbial eukaryotes are essential members of the Lake Cadagno chemocline because of autotrophy and their interactions with phototrophic sulfur bacteria via C, N, and S metabolism. This work presented the first near-complete genome (including nuclear and chloroplast assemblies) of a novel green algae species related to *Chlorellaceae*, providing genomic and phylogenomic evidence for this overlooked microbial eukaryote in Lake Cadagno. Based on this genomic work, primers can be designed to monitor the seasonal abundance of *Chlorella*-like microorganisms and morphologically characterize them using microscopy. Notably, *Chlorella*-like species thrive in almost no light and persist in anoxia; thus, its carbon fixation metabolism and genes for heat shock proteins may warrant its potential for biotechnological applications. Additionally, the chloroplast genomes of *Chlorella*-like and *Cryptomonas*-like species indicated that both microorganisms are capable of in situ oxygenic photosynthesis, a process that has been previously proposed in the anoxic waters of Lake Cadagno [19]. However, the similarities between *Cryptomonas* and cyanobacteria containing phycobilisomes suggest caution when reporting cyanobacteria in Lake Cadagno using flow cytometry [12, 21, 22].

## Materials and methods

### Sample collection and DNA extraction for shotgun DNA metagenomics

Samples were collected from Lake Cadagno, situated at an altitude of 1921 m above sea level (46.5504 °N, 8.7119 °E) in the Swiss Alps. The sampling strategy has been described in a previous study [16]. Briefly, 20 L of water was collected between two subsequent days, 28–29 August 2017, from the stratified zones of Lake Cadagno. The upper-oxic mixolimnion (5, 9, and 11 m) was sampled on the 28th of August (day 1), the chemocline (13, 15, and 15.5 m), and the monimolimnion (17 m) was sampled on day 2 (29th of August). The collected water samples were pre-filtered using a 55 µm mesh to remove zooplankton [14] and subsequently passed through a filtration setup equipped with 0.22 µm-filters (cat. #GPWP14250 142 mm Express Plus filter, Millipore, Darmstadt, Germany). After filtration, the 0.22 µm-filters were flash-frozen at −196 °C and stored at −80 °C until DNA extraction, performed in October 2018, as explained previously [16]. An additional whole water sample without a mesh (15-w) was collected from the turbidity peak.

### DNA sequencing and metagenome assembly

**Fig. 8:**
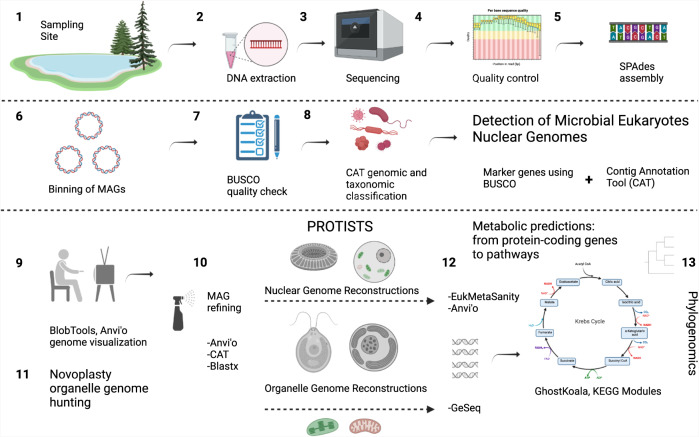
Overview of metagenomics pipeline to study microbial eukaryotes. Step-by-step guide to eukaryotic metagenomics pipeline (created with Biorender.com).

PCR-free libraries were prepared with a read length and insert size of 250 bp and sequenced using HiSeq 4000 (Illumina, San Diego, CA, USA) at the University of Michigan Advanced Genomics sequencing core facility. A step-by-step guide on microbial eukaryote genome hunting is available at GitHub (https://github.com/JSSaini/Pipeline_For_Lake_Cadagno_Eukaryotic_Metagenomics; Fig. 8). In summary, raw reads were trimmed, quality checked, and normalized using BBDuk and BBNorm in BBTools (v38.00) [63]. These normalized reads were assembled using SPAdes (v3.15.0) at default kmer lengths of 21, 33, and 55 using 16 CPUs and 250GB of memory [64]. Metagenomics mode (*--meta*) was used for all samples except 15-w, for which the assembly procedure was successful (i.e., analysis run to completion) without using the “*--meta*” flag. The contig names of each assembly were simplified using Anvi’o (*anvi-script-reformat-fasta*, v7), and contigs less than 1,000 nucleotides (*-l* 1,000) were removed [65].

Raw reads were mapped to the metagenome assemblies (including nuclear and chloroplast) using Bowtie 2 (v2.4.2) [66] and SAMtools (v1.12) [67]. The resulting binary alignment map (BAM) was sorted and indexed using Anvi’o (*anvi-init-bam*, v7) [65]. Contigs were then binned into MAGs using CONCOCT (clustering contigs with coverage and composition, v1.1.0 [29]) with default parameters using two strategies: by providing coverage information from (a) the individual library and (b) from all libraries, referred to as non-competitive and competitive binning, respectively.

### Obtaining representative Chlorophyta MAG from the chemocline

Chlorophyta MAGs with >90% BUSCO (v5.2.0) completeness were detected in all four chemocline samples (13, 15, 15-w, and 15.5 m) using competitive and non-competitive binning. To check for similarity, these MAGs were compared based on average nucleotide identity (Fig. S5) using dRep (v2.2.3) [68]. Chlorophyta-specific reads were extracted by mapping raw reads using Bowtie 2 (v2.4.2) and SAMtools (v1.12) to the concatenated Chlorophyta MAGs from the non-competitive binning to obtain the maximum number of Chlorophyta reads. Next, the Chlorophyta-specific raw reads (R1 and R2) were extracted from the resulting BAM files using *bamtofastq* (v1.1.0). SPAdes (v3.15.0) [64] coassembled Chlorophyta-specific raw reads at a default kmer length of 21, 33, and 55 nucleotides using two strategies. In the first strategy, SPAdes coassembled Chlorophyta-specific raw reads from all four samples (13, 15, 15-w, and 15.5 m). And in the second strategy, Chlorophyta-specific raw reads from only two samples were coassembled (13 and 15.5 m). The latter assembly was prioritized for rebinning because of the higher N50 value.

The contig names of each assembly were simplified using Anvi’o (*anvi-script-reformat-fasta*, v7), and contigs less than 2500 nucleotides (*-l* 2,500) were removed [65]. The Chlorophyta-specific assembly was rebinned using CONCOCT [29] (v1.1.0), providing coverage information (BAM) from all four chemocline samples.

### Classification, quality assessment, refinement, and visualization of MAGs

#### Genomic and taxonomic classification

Raw MAGs were classified using the contig and bin annotation tool (CAT and BAT, v5.2.3) [69]. Gene prediction in CAT/BAT was performed using prodigal [70]. The predicted open reading frames (ORFs) were queried to the NCBI non-redundant protein database (updated 24 July 2021) using DIAMOND (v0.9.14.115) [71–73]. To obtain an overview of the eukaryotic community composition, eukaryotic 18S rRNA gene sequences were extracted from metagenomics reads using phyloFlash (v3.4) [74]. The composition of the prokaryotic community based on 16 S amplicon gene sequencing was presented in a previous publication [16].

#### Quality assessment

MAGs were assessed with BUSCO (v5.2.0) using the “*--auto-lineage”* mode [75, 76]. BUSCO relies on a collection of single-copy orthologs generated from OrthoDB v10 [77] to identify complete, duplicated, fragmented, and missing single-copy genes. With the “*--auto- lineage”* mode, BUSCO attempts to identify the most suitable dataset for the assessment and allows the analysis of both prokaryotic and eukaryotic MAGs.

#### Bin refinement

MAGs were refined by referring to the GC content and coverage using the Anvi’o interface (*anvi-refine*, Fig. S6) [65]. In addition, the Chlorophyta MAG refinement was guided by CAT taxonomy [69], which aided in removing potential contaminants.

#### Visualization

Statistics on eukaryotic MAGs (size, contig length, GC content, and coverage) were visualized using BlobToolKit (v2) [78], which uses Diamond BLASTx for taxonomic classification (v0.9.14.115) (nr database, updated 24 July 2021) [71–73, 79].

### Gene prediction, quantification, and functional annotation of microbial eukaryotes

Protein-coding genes in eukaryotic genomes were predicted using EukMetaSanity (v0.1.0) [80] based on AUGUSTUS (v3.4.0) [81] and MetaEuk (v34c21f2bf34c76f852c0441a29b104e5017f2f6d) [82] gene predictors. The mean depth of gene coverage was calculated using Bowtie 2 (v2.4.2) [66], SAMtools (v1.12), and Anvi’o (v7). The identified protein-coding genes were mapped against the OrthoDB database at the Trebouxiophyceae level (which includes 5 species, v10.1) using the online mapping and charting tool [38, 83], and also mapped at the Chlorophyta level (which includes 17 species, v11) using the OrthoLoger (v3.0.3) software [83]. Additionally, the proteome was mapped to eggNOG (v5.0) orthology resource [84] using eggNOG-mapper (v2.1.9) [85]. KEGG orthology (KO; or K numbers) of the respective genes were mapped to KEGG pathways using the codes provided in the R script at Github under code availability. Optionally, GhostKOALA (v2.2) was used to visualize KEGG pathways based on the KEGG mapper (v5) [86–88].

### Comparative phylogenomics and quality assessment of Chlorellaceae genomes

We performed a phylogenomic analysis to phylogenetically place the newly discovered Chlorophyta species. The assemblies of related species belonging to Chlorellaceae (NCBI TaxID:35461) were retrieved from NCBI and are listed in the supplementary table (Table S10). *Chlamydomonas reinhardtii* (GCA_000002595.3) was used as the outgroup. To construct the phylogenomic tree, this study followed a slightly modified version of the snakemake workflow described by Manni et al. 2021 [89], relying on BUSCO [75, 76] to find and extract single-copy orthologs to infer phylogenies. BUSCO (v5.2.2) was run in genome mode (BUSCO_Metaeuk workflow) on each genome assembly using the chlorophyta_odb10 dataset (1,519 markers). The identified single-copy genes that were shared across 100% of the species (with no duplicates across all species) were extracted. For each orthologous group, proteins were aligned using MAFFT (v7.505) [90] and trimmed using trimAl (v1.4 rev15) [91]. The single alignments were concatenated with AMAS (v1.0) [92], and the resulting super-alignment was used to infer a maximum likelihood phylogeny with IQ-TREE (v2.1.2) [93]. The phylogenetic tree was visualized using Dendroscope (v3.7.6) [94] and annotated using ggtree (v3.0.1) [95] in R-studio [96] (v1.4.1106) and Adobe Illustrator (v25.2.1). The results from BUSCO were also used to compare the quality of the newly assembled *Chlorella*-like MAG with the available *Chlorellaceae* genomes deposited at NCBI.

### Organelle hunting and chloroplast phylogenomics

Chloroplast contigs were identified using BLASTn to obtain the best hits (*-max_target_seqs 1*) for each query sequence. *P. kessleri* (NC_012978.1; *Chlorella*) and *G. theta* (NC_000926.1; Cryptophyceae) chloroplast genomes were used as queries. The two identified tentative chloroplast genomes (contigs c_000000000152_15mw and c_000000000134-15mm) were termed chloroplast A (cpA) and chloroplast B (cpB), and were used as templates for circularization via NOVOPlasty [97] with a genome size range set to 80,000–200,000 *nt* and the default kmer length of 33 nucleotides.

Chloroplast phylogenomics was performed using the prospective chloroplast genomes of Lake Cadagno (cpA and cpB) with an additional list of chloroplast genomes from the following three families: (1) Chlorellaceae [Taxonomy ID:35461], (2) Cryptophyceae [Taxonomy ID:3027], and (3) Stramenopile [Taxonomy ID:33634]. Stramenopile chloroplasts were used as the outgroups.

The accession numbers of the chloroplast genomes used in the phylogenomic analysis are provided in supplementary (Table S11). Eighteen marker genes were used to construct the chloroplast phylogenetic tree, including ATP synthase (*atpA*, *atpB*, and *atpC*), large ribosomal subunits (*rpl2*, *rpl5*, *rpl12*, *rpl14*, *rpl19*, and *rpl23*), small ribosomal subunits (rps3, rps8, rps9, and rps19), photosystem I (psaC), and photosystem II (*psbA*, *psbB*, *psbE*, and *psbH*). These markers were individually aligned using MAFFT (v 7.487) [98], followed by quality assessment and removal of ambiguous sequences using Gblocks (v0.91b) [99]. Phylogenomic tree inferences were made using MEGA (v11.0.10) [100] by selecting the maximum likelihood using the Jones-Taylor-Thornston (JTT) method and bootstrapping (*n* = 100). The tree was visualized using Dendroscope (v3.7.6) [94] and annotated using ggtree (v3.0.1) [95] in R-studio [96] (v1.4.1106) and Adobe Illustrator (v25.2.1).

### Metagenome assembly and abundance statistics

Statistics, including size, GC content, number of contigs, N50 and L50 values of metagenomic assemblies, were calculated using *stats.sh* (individual) and *statswrapper.sh* (multiple) scripts in BBMap (v38.96) [101]. The N50 metric is the length of the shortest contig for which half of the genome is assembled on contigs of length N50 or longer, and the L50 value is the minimum number of contigs required to reach 50% of the genome assembly. The mean depth of coverage, number of reads, and relative abundance of purple and green sulfur bacteria and eukaryotic algae MAGs were calculated using Bowtie 2 (v2.4.2) [66], SAMtools (v1.12), Anvi’o (v7), and CoverM (v0.6.1) [102].

## Supplementary information

Supplementary figures and table legends only

Supplementary tables excel workbook

## Data Availability

Raw reads are available at the NCBI under the sequence read archive SUB11916861 and under the accessions SRR21025699, SRR21025700, SRR21025701, and SRR21025702. The GenBank ID for the *Chlorella*-like MAG is JAOAOU000000000.1. The assembled contigs from raw reads, MAGs, and other data were deposited in Zenodo (https://zenodo.org/record/7505505) [103].

## Code availability

A step-by-step guide to the eukaryotic metagenomics pipeline has been made available on GitHub, including the R script used to generate figures (https://github.com/JSSaini/Pipeline_For_Lake_Cadagno_Eukaryotic_Metagenomics).
