# Supplementary material for: Genomic insights into the coupling of a Chlorella-like microeukaryote and sulfur bacteria in the chemocline of permanently stratified Lake Cadagno
Source: ISME J. 2023 Apr 8;17(6):903–15. doi: 10.1038/s41396-023-01396-y (PMC10203261; doi:10.1038/s41396-023-01396-y)
Supplement: Supplementary file 1 — Supplementary figures and table legends only [file 41396_2023_1396_MOESM1_ESM.docx]

# ***Supplementary information for***

**Genomic insights into the coupling of a *Chlorella*-like microeukaryote and sulfur bacteria in the chemocline of permanently stratified Lake Cadagno**

Jaspreet S Saini^1,2,3,4#^, Mosè Manni^3,4^, Christel Hassler^2,5^, Rachel Cable^6^, Melissa B. Duhaime^6^, Evgeny M. Zdobnov^3,4#^

^1^Current address: Laboratory for Environmental Biotechnology, Ecole Polytechnique Fédérale de Lausanne, Lausanne, Switzerland

^2^Department F.-A Forel for Environmental and Aquatic Sciences, Earth and Environmental Sciences, University of Geneva, Switzerland

^3^Department of Genetic Medicine and Development, University of Geneva, Switzerland

^4^Swiss Institute of Bioinformatics, Geneva, Switzerland

^5^Institute of Earth Sciences, University of Lausanne, Switzerland

^6^Department of Ecology and Evolutionary Biology, University of Michigan, Ann Arbor, MI, United States

**Correspondent footnote**

| Dr. Jaspreet Singh Saini  EPFL ENAC IIE LBE  CH C3 425 (Bâtiment CH), Station 6  CH-1015 Lausanne  Telephone +41 (0) 216934721  [jaspreet.saini@epfl.ch](mailto:jaspreet.saini@epfl.ch) | Prof. Evgeny Zdobnov  [Computational Evolutionary Genomics](https://sib.swiss/evgeny-zdobnov-group)  CMU / 9022  Geneva 4  Telephone +41 22 379 59 73  [evgeny.zdobnov@unige.ch](mailto:evgeny.zdobnov@unige.ch) |
| --- | --- |

Number of supplementary tables: 11 (Attached in separate excel workbook).

Number of supplementary figures: 6

**Supplementary table legends. Tables provided in excel workbook.**

**Table S1:** Statistics on normalized and quality control reads collected from the four samples of Lake Cadagno chemocline.

**Table S2:** Summary of MAGs obtained from four chemocline samples using competitive binning through CONCOCT. MAGs were classified and quality assessed using the CAT/BAT and BUSCO.

**Table S3:** Summary of MAGs obtained from four chemocline samples using non-competitive binning through CONCOCT. MAGs were classified and quality assessed using the CAT/BAT and BUSCO.

**Table S4:** Summary of representative Chlorophyta MAG from Lake Cadagno after manual MAG refinement using Anvi’o.

**Table S5**: CAT taxonomy on prospective contaminants removed from Chlorophyta MAG during manual bin refinement.

**Table S6:** Summary of representative Chlorophyta MAG (refined) obtained from the chemocline of Lake Cadagno.

**Table S7:** OrthoLoger-based functional annotations of protein-coding genes of *Chlorella*-like MAG.

**Table S8:** eggNOG-based functional annotations protein-coding genes of *Chlorella*-like MAG.

**Table S9:** Collection of protein-coding genes involved in C, N, and S metabolism of Chlorella-like MAG annotated using eggNOG and eggNOG-mapper. Only genes belonging to the Chlorophyta and Viridiplantae taxonomy were used.

**Table S10:** Assembly accessions of the 32 Chlorecellaceae (TaxID: 35461) species from NCBI which were used to construct the phylogenomic tree in Fig. 3.

**Table S11:** The 19 chloroplast assemblies from NCBI, which were used to construct organelle phylogenetic tree in Fig. 5.

**Supplementary figures and legends.**

Figure S1


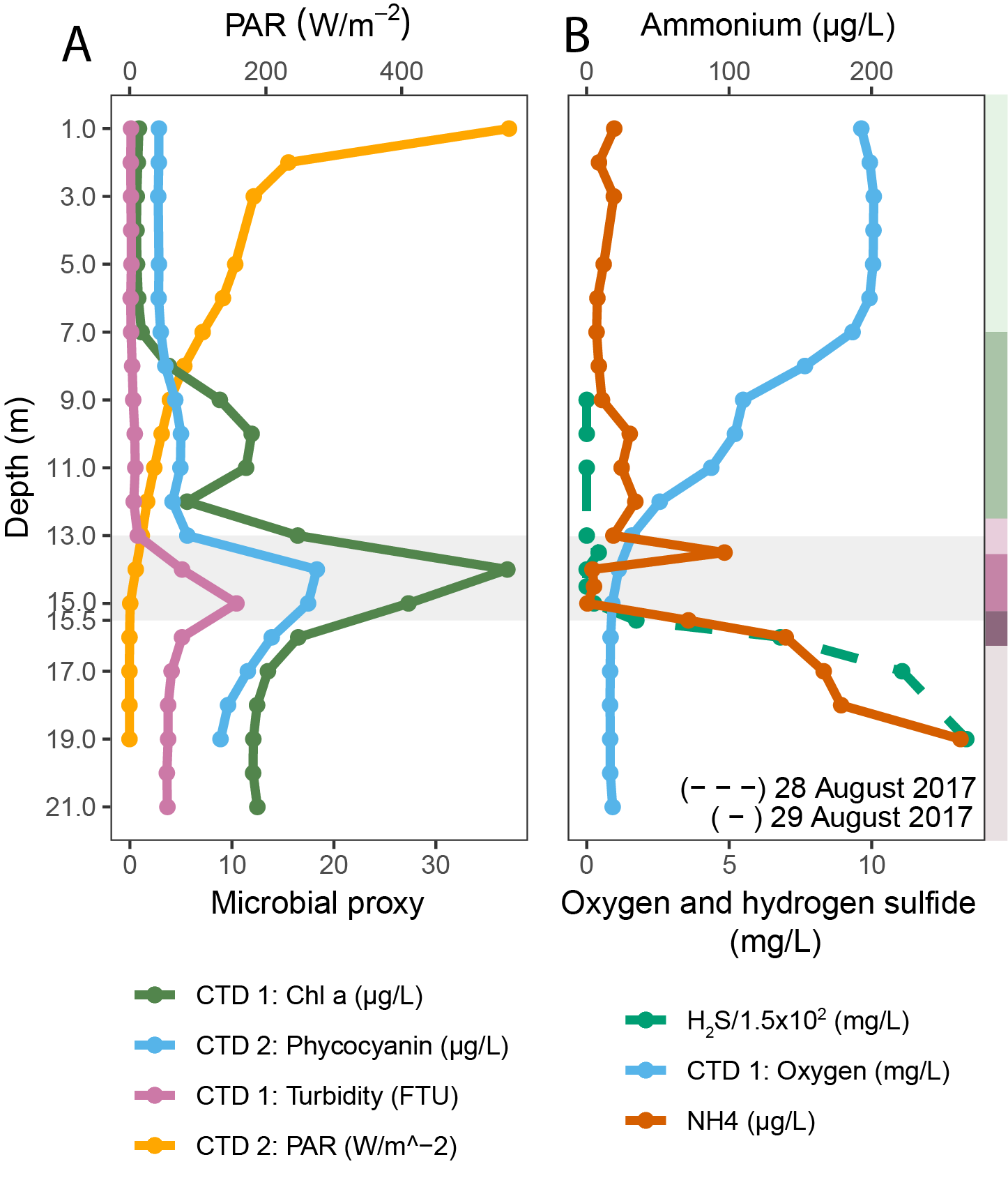


**Figure S1:** Physicochemical parameters of Lake Cadagno water-column collected on the days of metagenomics sampling. CTD 1 and CTD 2 data were adapted from the previous publication [^16^](https://paperpile.com/c/zzYIX8/GwH54), where the position of the chemocline was between 13-15.5 m, indicated by a light gray zone. The light-green, dark-green, light-lilac, lilac, dark-lilac, and light-brown color columns on the right y-axis indicate the zone of high-O_2_ mixolimnion, medium-O_2_ mixolimnion, mixolimnion-chemocline transition zone, the chemocline, and the lower anoxic chemocline, and monimolimnion. (A) Chl *a* (dark green) and phycocyanin (light blue) are used for phytoplankton, and turbidity (pink) indicates a proxy for purple and green sulfur bacteria, whereas light (PAR) is a yellow line. (B) Chemical profiles of oxygen, hydrogen sulfide, and ammonium are represented by light green, light blue, and orange lines.

Figure S2


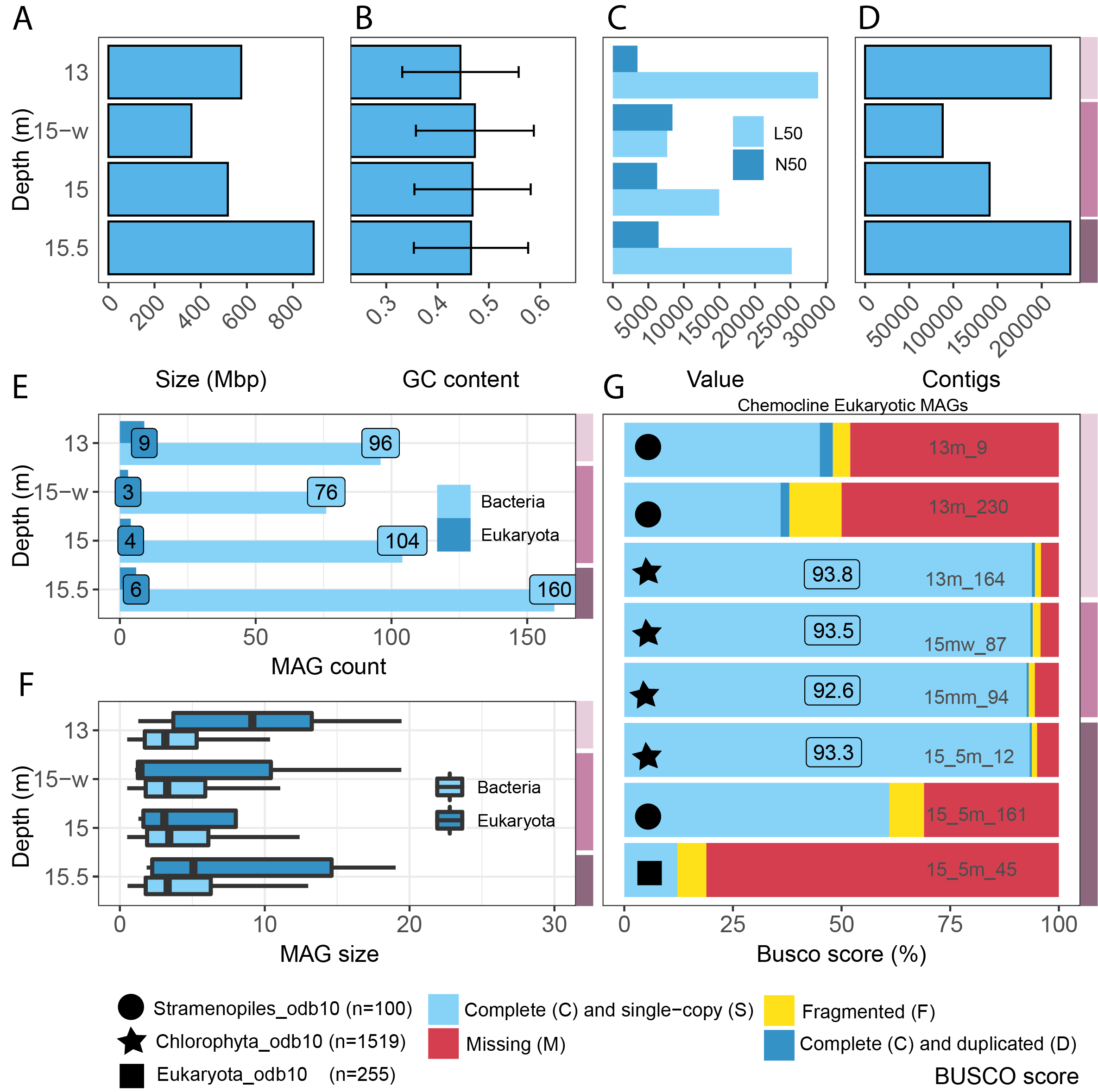


**Figure S2:** Overview of four Metagenomes collected between 13-15.5 m depth of Lake Cadagno’s chemocline, where 15-w represents a whole water sample (without 55 µm mesh). Panel A-D summarizes assembly statistics from SPAdes, including (A) size, (B) average GC content, (C) N50 and L50 values, (D) and number of contigs. (E-G) Non-competitive binning of bacterial and eukaryotic MAGs using CONCOCT, classified and quality assessed by CAT/BAT taxonomy (hits >0.5 bit-scores) and BUSCO (only eukaryotic MAGs with >10% complete). The light-lilac, lilac, and dark-lilac color columns on the right y-axis indicate a mixolimnion-chemocline transition, the chemocline, and the lower chemocline, respectively.

Figure S3


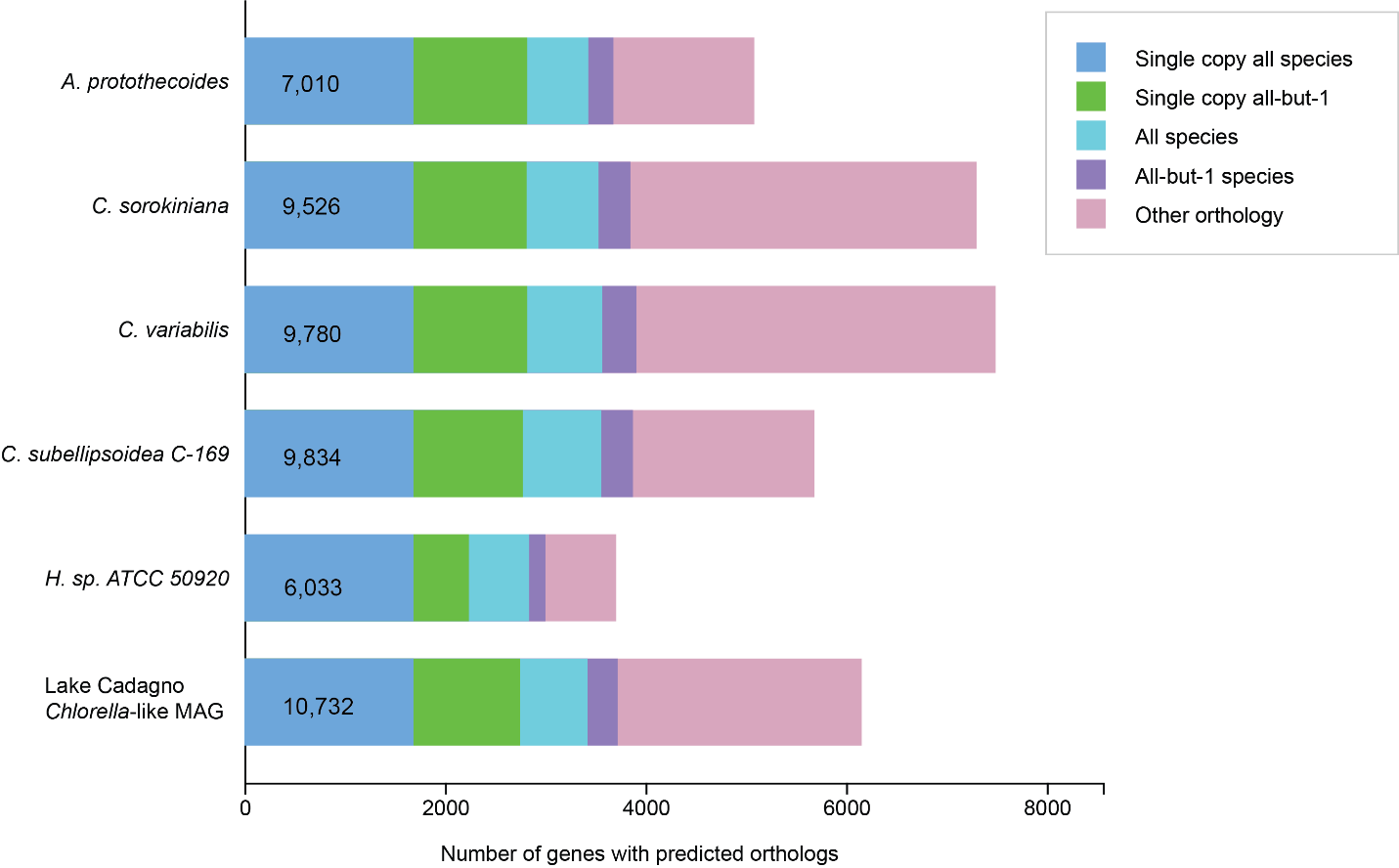


**Figure S3:** Orthology chart of *Chlorella-like MAG* and 5 other related species available in OrthoDB database. Genes with at least an ortholog in one of the other species are reported. Gene counts are grouped into the following categories: single-copy orthologs in all 6 species, single-copy in all except one species, genes present in all species, present in all except one species, and genes with other types of orthologous relationships. Values on bars correspond to total gene counts including genes with no detectable orthology.

Figure S4


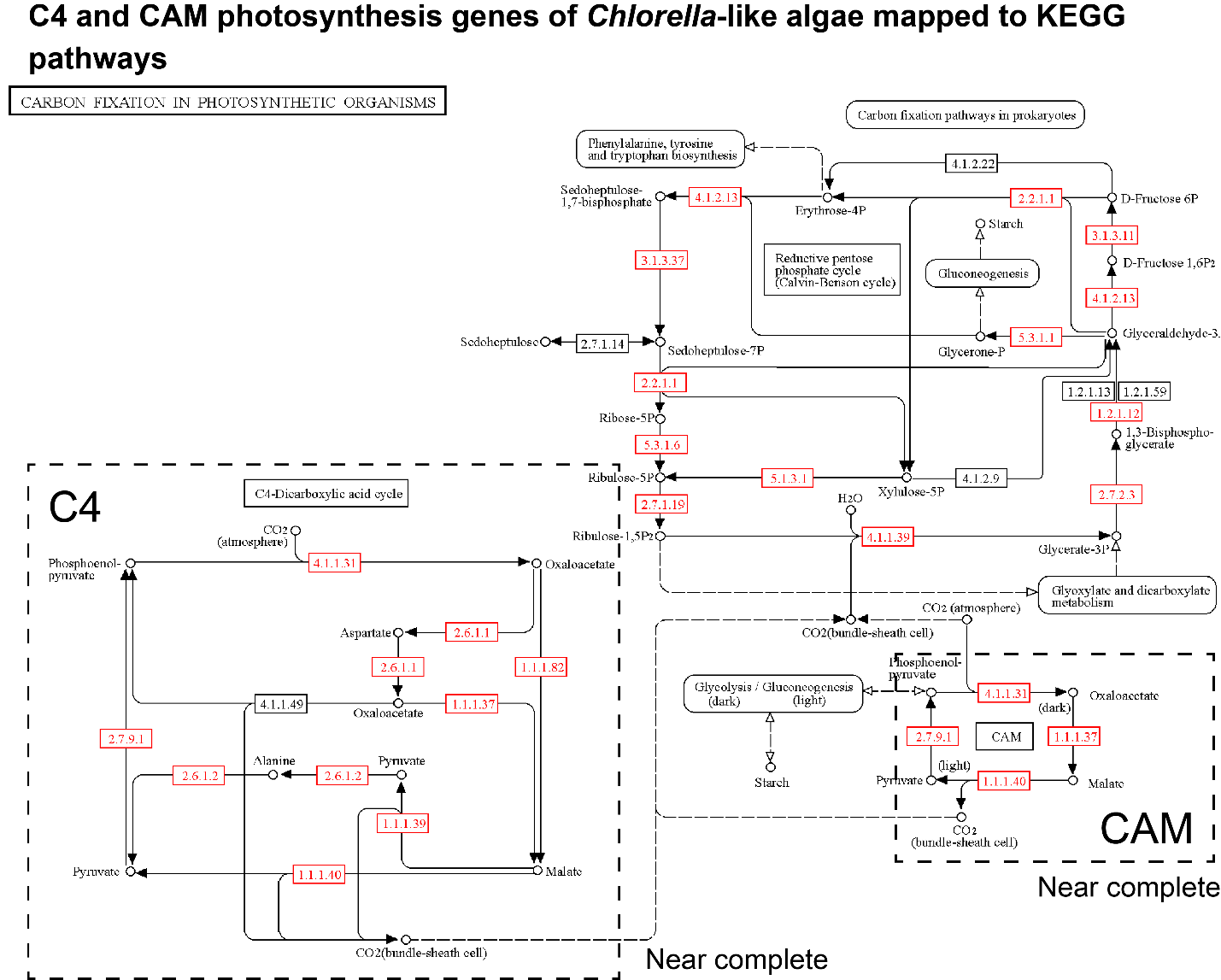


**Figure S4:** Carbon metabolism genes were mapped to KEGG pathways using orthologs (K numbers of pathway, ko00710, Table 2). To visualize carbon fixation pathways (ko00710), a list of respective K numbers is provided at <https://www.genome.jp/pathway>. This study identified K numbers in red in the MAG of *Chlorella*-like algae.

Figure S5


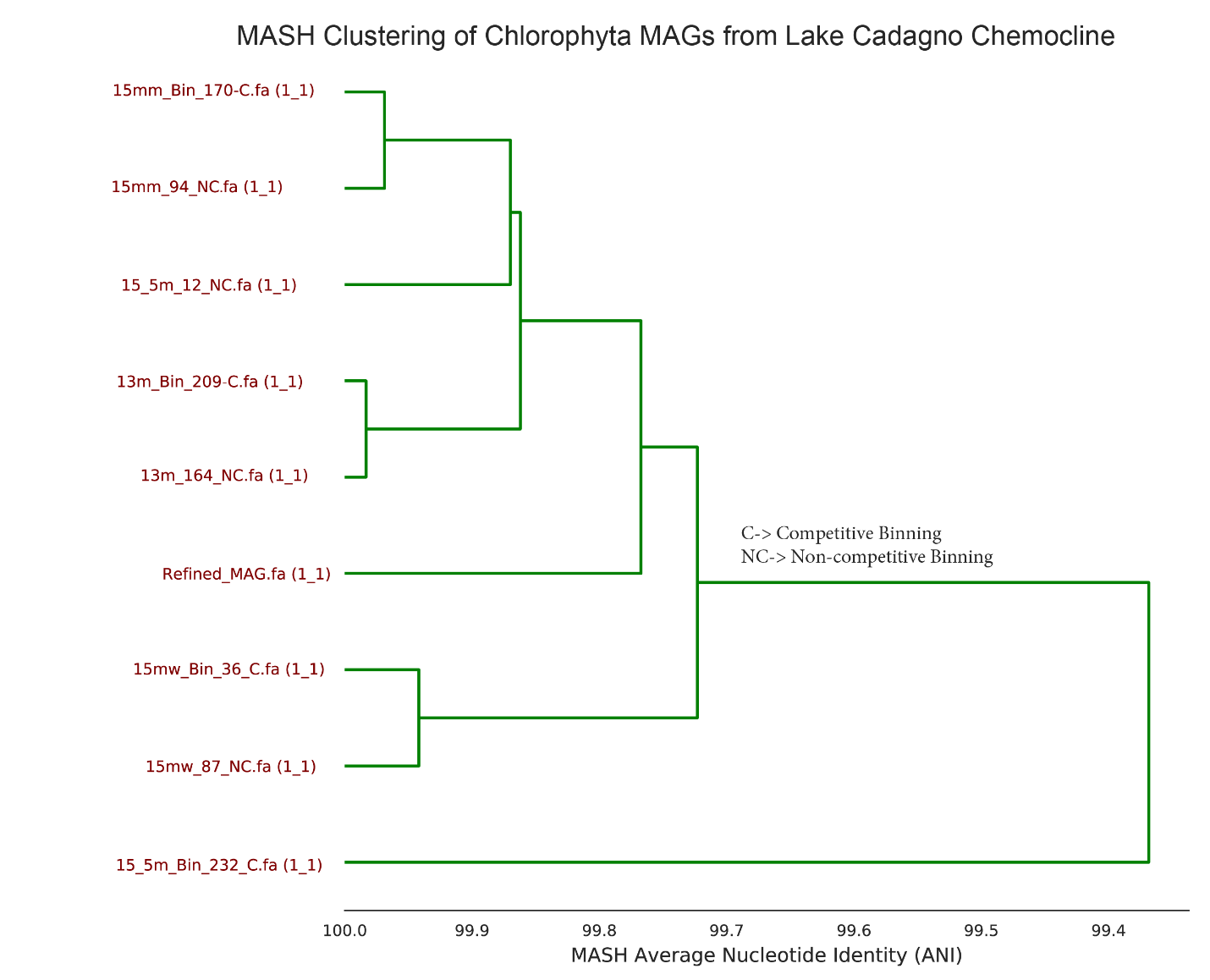


**Figure S5:** dRep-based MASH algorithm clustering of refined and unrefined Chlorophyta MAGs obtained using competitive and non-competitive binning.

Figure S6


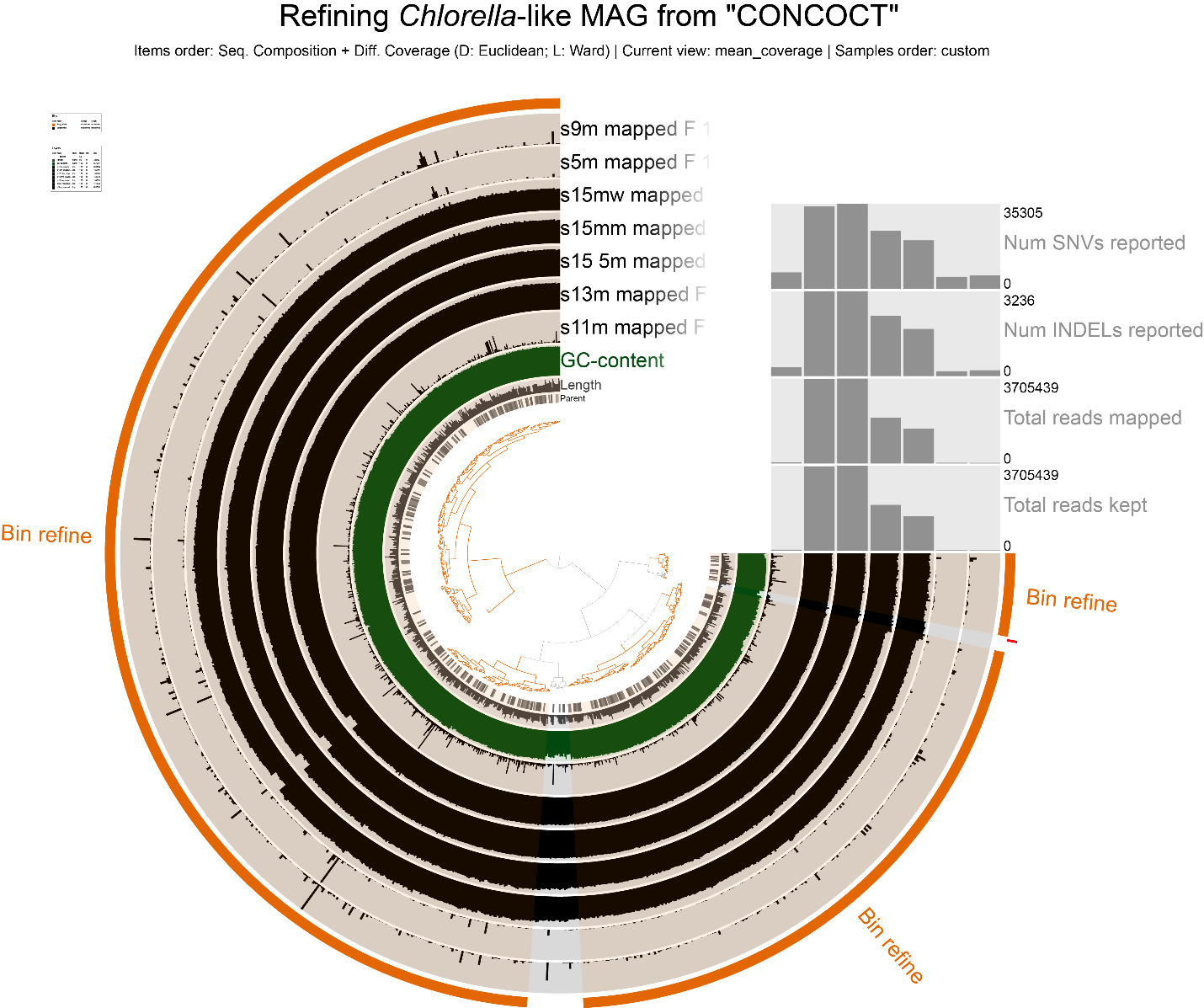


**Figure S6:** Snapshot of manual refining of Chlorophyta MAG using Anvi’o interface by using coverage and GC content.
